# The Effect of Different Bleaching Techniques Using 6% Hydrogen Peroxide: Penetration Inside the Pulp Cavity, Bleaching Efficacy and Toxicity

**DOI:** 10.1590/0103-644020245975

**Published:** 2024-12-06

**Authors:** Gabrielle Gomes Centenaro, Michael Willian Favoreto, Taynara de Souza Carneiro, Michel Wendlinger, Christiane Philippini Ferreira, Lívia Câmara de Carvalho Galvão, Alessandro Dourado Loguercio

**Affiliations:** 1 Department of Restorative Dentistry, School of Dentistry, State University of Ponta Grossa, PR, Brazil; 2 Department of Stomatology, IDIBO research group, Health Sciences Faculty, Rey Juan Carlos University, Alcorcón, Madrid, Spain; 3 Department of Chemistry, State University of Ponta Grossa, PR, Brazil; 4 Department of Postgraduate Program in Dentistry, CEUMA University, São Luís, MA, Brazil

**Keywords:** Hydrogen Peroxide, Dental Enamel Permeability, Tooth Bleaching

## Abstract

This in vitro study aimed to quantify the penetration of hydrogen peroxide (HP), bleaching efficacy (BE) and toxicity in larvae in different bleaching techniques using 6% HP. Sixty maxillary premolars were divided in six groups (n = 10): Pola Luminate (PL), VivaStyle Paint On PIus (VS), PolaDay (PD), White Class (WC) and Whiteness HP Automixx (AM). A group not exposed to bleaching agents was evaluated as a control group (CG). Previously, the initial HP concentration in the bleaching agents was determined via titration and pH was measured with a digital pH meter. The concentration (µg/mL) of HP inside the pulp cavity was assessed using UV-Vis spectrophotometry. The BE (ΔE^*^_ab_ and ΔE_00_) was evaluated with a digital spectrophotometer. The toxicity was evaluated in larvae model. Data from HP penetration and color change were evaluated for a one-way ANOVA and the Tukey’s test, as well as Kaplan-Meier death curve for toxicity (α = 0.05). VS, PD and AM had an initial concentration lower than that the manufacturers reported. VS had a more acidic pH. PL and WC showed a significantly lower HP amount inside the pulp cavity when compared to VS (p = 0.001). Regarding BE, no significant difference was observed for ΔE^*^_ab_ (p = 0.38), and ΔE_00_ (p = 0.42). No toxicity was observed when all 6% HP was compared to GC (p > 0.05). All 6% HP products tested showed bleaching efficacy, low penetration into the pulp chamber and were non-toxic to Tenebrio molitor larvae.

## Resumo

Este estudo *in vitro* teve como objetivo quantificar a penetração de peróxido de hidrogênio (PH), eficácia clareadora (EC) e toxicidade em larvas em diferentes técnicas de clareamento utilizando 6% de PH. Sessenta pré-molares superiores foram divididos em seis grupos (n = 10): Pola Luminate (PL), VivaStyle Paint On PIus (VS), PolaDay (PD), White Class (WC) e Whiteness HP Automixx (AM). Um grupo não exposto aos agentes clareadores foi avaliado como grupo controle negativo (GC). A concentração inicial de PH nos agentes clareadores foi determinada por meio de titulação e o pH medido com um pHmetro digital. A concentração (µg/mL) de PH no interior da cavidade pulpar foi avaliada por espectrofotometria UV-Vis. A EC (ΔE_ab_^*^ e ΔE_00_) foi avaliada com um espectrofotômetro digital. A toxicidade foi avaliada em modelo larval. Os dados de penetração de PH e mudança de cor foram avaliados por uma ANOVA de uma via e o teste de Tukey, bem como a curva de morte de Kaplan-Meier para toxicidade (α = 0,05). VS, PD e AM tiveram uma concentração inicial menor do que a relatada pelos fabricantes. VS teve um pH mais ácido. PL e WC apresentaram uma quantidade significativamente menor de PH dentro da cavidade pulpar quando comparados ao VS (p = 0,001). Com relação a EC, não foi observada diferença significativa para ΔE_ab_ (*p* = 0,38) e ΔE_00_ (*p* = 0,42). Nenhuma toxicidade foi observada quando todos os 6% PH foram comparados com GC (*p* > 0,05). Todos os produtos 6% PH testados apresentaram eficácia clareadora, baixa penetração na câmara pulpar e não foram tóxicos para larvas de *Tenebrio molitor*.

## Introduction

Tooth bleaching has received a lot of attention in recent years due to the dissatisfaction of patients with their tooth discoloration ^1^ ^,^ ^3^. Currently, techniques for vital tooth bleaching are consolidated in the literature: at home, where the patient, under a professional’s supervision, performs the procedure at home, using carbamide peroxide or hydrogen peroxide (HP) in lower concentrations; and in-office bleaching, which the dentist performs during clinical care using HP in higher concentrations (up to 40%) ^1^ ^,^ ^2^. For both protocols, several brands and commercial forms of bleaching gels are available on the market, and the professional chooses the best one that fits each patient, taking into account individual needs, bleaching efficacy and adverse effects ^2^.

However, the main drawback patients report is that bleaching induces tooth sensitivity ^3^. This occurrence is explained by the ability to penetrate the tooth structure due to its low molecular weight and reach the pulp chamber, thus promoting oxidative stress and damage to the pulp cells ^4^. Although, it is clear that the greater the amount of HP used, the greater the reported tooth sensitivity ^3^. Several studies showed high levels of tooth sensitivity even in at-home bleaching, mainly when higher concentrations or longer time tray wear are applied ^5^ ^,^ ^6^. This high concentration can result in patients stopping the treatment due to related sensitivity induced by tooth bleaching ^3^ ^,^ ^6^.

In view of this, several clinical protocols that the professional can use to try avoiding or at least reduce the tooth sensitivity are proposed, and using lower concentrations of the bleaching gel is an interesting alternative ^3^. Unfortunately, there is no consensus regarding the ideal HP concentration that provides bleaching efficacy associated with the lower level of tooth sensitivity. However, the Scientific Committee on Consumer Products of the European Union published a resolution that only approved products with up to 6.0% HP as safe to use ^7^.

Because of this, several manufacturers have introduced products to the market that exclusively fit this specific indication. Although all materials contain up to 6% HP, these products are available in different application forms: such as varnishes or syringes (over-the-counter products: OTC), at-home products (single syringe), and more recently, in-office bleaching products (dual syringes with self-mixing). The various formulations of HP 6% present different indications depending on the patient's needs and preference. Additionally, (OTC) products have emerged as a low-cost alternative for bleaching discolored teeth compared at home and in-office bleaching gels ^1^. Despite all materials evaluated containing the same HP concentration, various chemical differences among the products can impact the final results. For example, variations in pH level, pH stability, and viscosity directly affect the amount of HP released and, consequently, observed inside the pulp chamber ^8^ ^,^ ^9^. Additionally, significant differences between the labeled and the actual concentration of HP inside several products were previously observed ^10^ ^,^ ^11^. Therefore, all these factors can influence the final amount of HP inside the pulp chamber, providing the main justification for conducting the present study.

However, to the authors' knowledge, in vitro studies evaluating different forms of bleaching gels with 6% HP, applied under real-world conditions, have not yet been performed. In view of this, the objective of this in vitro study was to evaluate the penetration of (HP) inside the pulp chamber, bleaching efficacy, and toxicity using different bleaching techniques that use 6% HP. This includes comparing at-home bleaching gels and the over-the-counter technique of bleaching varnishes with the in-office bleaching gel. The initial concentration of HP and the bleaching agents’ pH were also measured. The null hypothesis to be tested is as follows: ^1^ there will be no difference in penetration HP inside the pulp cavity, ^2^ bleaching efficacy and ^3^ toxicity larvae model when comparing different bleaching techniques containing 6% HP. Specifically, we will compare at-home bleaching gels and the over-the-counter technique of bleaching varnishes with the in-office bleaching gel.

## Materials and Methods

## Ethics committee approval

This study was submitted to the ethics committee, which approved it under the agreement number (5.131.737).

### Selection of teeth and inclusion and exclusion criteria

Sixty sound, fist and second maxillary premolars were obtained from Human Teeth Bank. The teeth underwent analysis under a microscope with a microscope at 10× magnification (Lambda LEB-3, ATTO instruments, Hong Kong, China) to standardize the selection process. Teeth with superficial morphological changes or enamel cracks were excluded. Prior to experimentation, the teeth were analyzed using a digital spectrophotometer (VITA Easyshade Advance 4.0, VITA Zahnfabrik, Bad Säckingen, Germany) and teeth lighter than A_2_ and darker than A_3_ were excluded according to the spectrophotometer. In total, 30 A2 and 30 A3 teeth were selected, and a balanced distribution was performed, ensuring that each group had the same references (5 A2 and 5 A3).

To standardize vestibular thickness of the specimens, radiographs were taken (Timex 70C; Gnatus, Ribeirão Preto, SP, Brazil). Each radiograph was made with an exposure time of 0.5 seconds and a focus-object distance of 30 cm (70 kVp, 7 mA). The central X-ray beam was focused at a 90° angle on the tooth’s mesial surface. After exposure, the images were digitally obtained, and the buccal dental thickness was measured with the New IDA software (Dabi Atlante, Ribeirão Preto, SP, Brazil). Based on previous studies, teeth with buccal thickness on average around 3.2-3.3 mm were included ^8^ ^,^ ^9^.

### Experimental groups

A block randomization based on the initial color of the tooth was conducted to randomly allocate the sixty teeth into six groups (n = 10), following the bleaching protocol outlined below: 6% HP at-home technique with varnishes of self-applied brush Pola Luminate (PL; SDI, Victoria, Australia) and VivaStyle Paint On Plus (VS; IvoclarVivadent, Schaan, Liechtenstein), 6% HP at-home technique with a custom-tray PolaDay (PD; SDI, Victoria, Australia) and White Class (WC; FGM Dental Product, Joinville, SC, Brazil) and 6% HP in-office technique with brush Whiteness HP Automixx (AM; FGM Dental Product, Joinville, SC, Brazil). A group not exposed to bleaching agents served as the negative control group (CG).

### Sample size calculation

The primary outcome of this study was the amount of HP within the pulp chamber. According to a previous study, the penetration value of 6% HP (Whiteness HP Automixx; AM; FGM Dental Product, Joinville, SC, Brazil) was 0.033 µg/mL, with a standard deviation of 0.016 µg/mL ^8^ ^,^ ^9^ ^,^ ^11^. This study anticipated a 65% reduction in the amount of HP, equating to 0.011 µg/mL, when applying the 6% in-office bleaching gel for a shorter duration (experimental group). This 65% reduction threshold was derived from our previous research, where similar levels of reduction were consistently with significant differences in outcomes. ^9^ ^,^ ^12^ ^,^ ^13^. Using a bilateral test with an alpha of 5% and a power of 80%, 10 teeth were required in each experimental group (CG, AM, PD, PL, VS, and WC). The sample size was determined (six interventions) using G*Power software version 3.1 using F tests for ANOVA (https://www.psychologie.hhu.de/arbeitsgruppen/allgemeine-psychologie-und-arbeitspsychologie/gpower).

### Specimen preparation

The specimens were prepared according to previous studies in the literature ^8^ ^,^ ^9^ ^,^ ^11^. Briefly, the roots of the teeth were removed approximately three millimeters from the enamel-cement junction. The pulp tissue was removed, and the access to the pulp cavity was slightly expanded using a spherical drill, taking care not to touch the pulp cavity’s inner vestibular region. This was done with the objective that 25 μL of the solution could be introduced inside the pulp cavity using a micropipette. After preparation, specimens were randomized into groups and new radiographs were taken to verify the buccal thickness of specimens by groups measured.

### Initial color change

All groups’ baseline color was measured before starting the bleaching procedure. For all bleaching gels evaluated, this procedure was repeated one week after completing the treatments using a digital spectrophotometer (VITA Easyshade Advance 4.0, VITA Zahnfabrik, Bad Säckingen, Germany), the device was calibrated both before conducting the color measurements and between the different groups assessed. To measure the specimens’ initial color, guides were made with dense condensation silicone (Coltoflax and Cub Kit Profile, Vigodent, Rio de Janeiro, RJ, Brazil) ^8^ ^,^ ^9^. To standardize the spectrophotometer’s position, a window with 6 mm diameter was made with a metal device in the middle one third of the buccal surface for each specimen in which the tip of the spectrophotometer was inserted. During this period, the specimens were immersed in artificial saliva (Pharmacy Eficácia, Ponta Grossa, PR, Brazil) containing carboxymethylcellulose, sodium chloride, potassium chloride, magnesium chloride, dibasic calcium phosphate, glycerin, xylitol, and distilled water. The solution was refreshed daily and kept at a constant temperature of 37ºC. The color parameters (L*, a* and b*) were recorded through the tip of the device inserted in the silicone guide. All the color evaluation was performed by one blind calibrated evaluator with superior color discrimination competency according to ISO/TR 28642 ^14^ and an intra-examiner agreement level of agreement of at least 85% when evaluated in two different moments (Kappa statistic) assessed color outcomes. The recordings were made in the same room with the same lighting conditions, with hydrated teeth.

### Obtaining the study calibration curve

The study used analytical products without prior purification and all solutions were prepared using deionized water. Initially, a standard analytical curve was drawn from a 5.000 μg/mL stock solution prepared from a concentrated solution (50% HP, Pharmacy Eficácia, Ponta Grossa, PR, Brazil). This solution was diluted in an acetate buffer solution (pH = 4) and titrated using traditional methods. The solution was titrated with a potassium permanganate solution to determine the analytical grade and the solution’s actual concentration ^8^ ^,^ ^9^ ^,^ ^11^. Based on this initial concentration, serial volumetric dilutions of 0.000-0.403 μg/mL were performed to draw the analytical curve. The known concentrations of HP were obtained using a Cary UV-Vis 50 spectrophotometer (Varian, Palo Alto, CA, USA). This procedure yielded a standard reference line for the extrapolation of the study samples’ results (R = 0.992; not shown data).

### Treatment bleaching protocols

**Figure 1:**
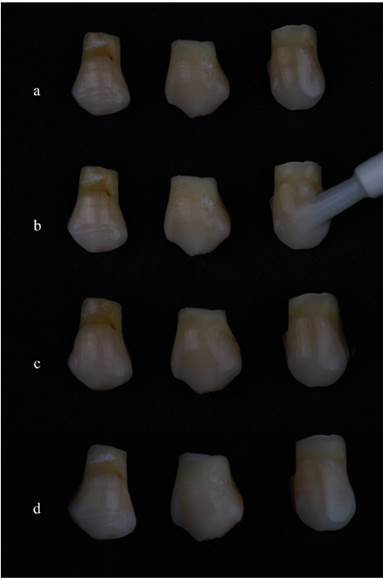
(A) Initial color; (B) Application of Pola Luminate (PL; SDI, Victoria, Australia) to the specimens; (C) During the bleaching procedure; (D) Final image after a bleaching session.

For all treatment bleaching protocols, a single calibrated and experienced operator was responsible for applying the materials. The bleaching agents were applied, according to the manufacturer's recommendations, in the vestibular enamel area and applied until it completely covered the buccal area of the teeth to be whitened (Box 1 and Figure 1). The application period of the bleaching gels PL, PD and WC were 30 minutes per day. VS was applied for 10 minutes per day, and AM was applied for 50 minutes per session. For the at-home bleaching protocol, the bleaching gels were applied for 14 days, and for in-office bleaching (AM) in 3 sessions, 50 minutes each with intervals of 7 days between them. The bleaching gel was removed with gauze and by carefully washing with deionized water only on the vestibular surface. The CG was kept out of contact with bleaching agents. Due to the potential contamination with HP, it is consistently utilized as a control group in studies evaluating the amount of HP inside the pulp chamber-a “negative control group” without the application of a bleaching agent ^8^ ^,^ ^9^ ^,^ ^11^. Box 1 describes more details of the application method for each material and Figure 1 provides additional details regarding the bleaching procedure.

**Box 1:**
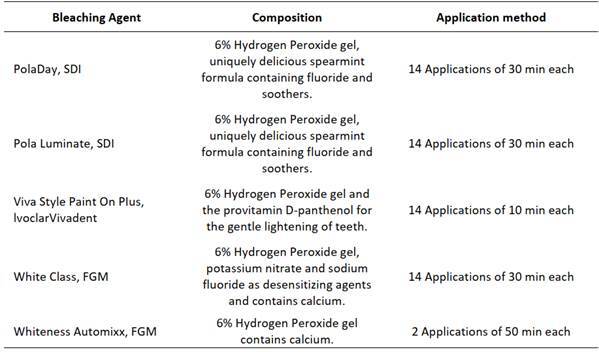
Commercial bleaching gel used in the study (manufacturer, composition and application method).

### HP concentration and pH measurements

To confirm the information that the manufacturers reported, the bleaching agents were submitted to evaluate the HP concentration and pH. The bleaching gels used in the study were titrated with a standardized potassium permanganate solution before the bleaching procedure, as described in the literature ^8^ ^,^ ^9^ ^,^ ^11^. This was done to determine the initial HP concentrations in the bleaching agents and to compare them with the information the manufacturer provided. The pH was measured as described in the previous literature ^9^ ^)^ using a digital pH meter (Extech pH 100: ExStik pH Meter; Extech instruments, Nashua, New Hampshire), and the data obtained were compared to those the manufacturer reported. For both measurements, data were performed in triplicate.

### HP quantification inside the pulp cavity

Following the bleaching procedure, the acetate buffer solution in each sample’s pulp cavity was removed in time immediately after the end of the bleaching procedures. The removed solutions were transferred to a glass tube. To remove the HP completely, this procedure was repeated with cleaning each tooth’s pulp cavity four times with 25 μL of the acetate buffer. This solution was transferred to the same glass tube. Sequentially, 100 μL of 0.5 mg/mL (Leucocrystal Violet, Sigma Chemical Co., St Louis, MO, USA) and 50 μL of 1 mg/mL of horseradish peroxidase (Peroxidase Type VI-A, Sigma Chemical Co., St. Louis, MO, USA) were added to the glass tube, along with deionized water (2.725 μL). This sequence was repeated separately for each tooth at different times. The resulting solution was measured using a Cary 50 UV-Vis spectrophotometer (Varian, Palo Alto, CA, USA). According to the Beer Law, absorbance directly corresponds to concentration. Therefore, the HP concentration (μg/mL) was determined by comparing it with the calibration curve already obtained.

### Final color change evaluation

All groups’ final color was measured one week after completing the treatments using a digital spectrophotometer (VITA Easyshade Advance 4.0, VITA Zahnfabrik, Bad Säckingen, Germany), and this measurement was considered final for all groups. It was performed in the midpoint of the buccal surface of the specimens, according to the standardized position previously described. The color parameters (L*, a* and b*) were recorded through the tip of the device inserted in the silicone guide. The L* value represented the lightness (the values ranged from 0 for black to 100 for white), the a* value represented the green-red coordinate (−a* green and +a* red) and the b* value represented the blue-yellow coordinate (−b* blue and +b* yellow). The color change before (baseline) and after one week of the treatments was given by the difference between the colors measured with the spectrophotometer using the CIELAB formula ^14^: ΔE^*^_ab_ = [(∆L*)^2^ + (∆a*)^2^ + (∆b*)^2^]^1/2^. Additionally, the color change was also calculated using an improved CIEDE 2000 formula ^15^, employs the same color parameters as the previous formula (L*, a* and b*), where L’ = L*; C’ = [a’ + b’]½ and h’ = tan -1 (b’/a’), CIEDE 2000: ∆E_00_ = [(ΔL/K_L_S_L_)^2^ + (ΔC/K_C_S_C_)^2^ + (ΔH/K_H_S_H_)^2^ + R_T_ (ΔC/ K_C_S_C_)(ΔH/ K_H_S_H_)]^1/2^. Where ΔL', ΔC', and ΔH' are differences in lightness, chroma, and hue, respectively; R_T_ is a rotation function that explains the interaction between chroma and hue in the blue region; S_L_, S_C_, and S_H_ are weighting functions, and K_L_, K_C_, and K_H_ are parametric factors adjusted for 1. ^16^

### Systemic toxicity of HP in Tenebrio molitor (T. molitor) larvae

Larval stage insects were used for the 6% HP toxicity assays. This choice was made by the authors because cytotoxicity tests described according to the ISO 10993-12:2012 (E) recommendations are more complicated and expensive compared to the present assay. Six groups of 20 *Tenebrio Molitor (T. molitor)* larvae (São Luís Biofactory Ltda, São Luís, MA, Brazil) weighing 70-120 mg and with no signs of melanisation were randomly selected and used for subsequent inoculation. According to the HP concentration values inside the pulp cavity for each group obtained in the previous test, two solutions were prepared: the minimal initial concentration, meaning, the exact concentration of HP values inside the pulp cavity for each group; and 10 times the minimal initial concentration of HP for each group. For this purpose, a trained operator injected 5 µL aliquots of solutions prepared into the haemocoel of each larva through the last left proleg using a 50 µL Hamilton syringe, the test was done in triplicate ^17^. After injection, larvae were stored at 37 ° C temperature, and the appearance of melanisation signs and survival were recorded at selected intervals for 7 days. Larvae were classified as dead if they were unable to move in response to touch.

### Statistical analysis

The data were analyzed using the Kolmogorov-Smirnov test to assess whether they were normally distributed and via the Barlett test for equality of variance to verify the assumption of equality of variances (not shown data). As the data showed normality, the data of the amount of the HP concentration (µg/mL) detected inside the pulp cavity, the initial concentrations of HP in the bleaching agents (%) and pH, as well as the bleaching pattern (ΔE^*^_ab_ and ΔE_00_) were subjected to two statistical evaluations: 1) one-way ANOVA and Tukey’s post-hoc test to compare different bleaching techniques and 2) one-way ANOVA and Dunnet’s post-hoc test to compare the values obtained in different bleaching techniques with those of the CG. Kaplan-Meier death curves were plotted and estimates of differences in survival were compared using a log-rank test to test the systemic toxicity of HP in larvae. Statistical significance was set at α = 0.05 (SPSS version 22.0, IBM Corp., Armonk, NY, USA).

## Results

### CHP concentration and pH measurements

Table 1 shows the initial concentrations of HP and pH measurements in the bleaching gel available. Regardless of the different bleaching techniques evaluated, a significant difference was observed between experimental groups (Table 1; *p* = 0.001). PL and WC showed similar and a significantly higher amount of HP when compared to VS, PD and AM, which showed similar results among them (Table 1; *p* > 0.05).

Regarding the pH measurements in the bleaching gel, regardless of the different bleaching techniques evaluated, a significant difference was observed among groups (Table 1; *p* = 0.001). AM showed a statistically higher (alkaline) pH, which was significantly different when compared to all other experimental groups (Table 1; *p* = 0.001). An intermediary group was observed for PL, PD and WC with a pH range between 5.5 and 5.9. On the other side, VS showed a statistically lower (more acidic) pH, which was significantly different when compared to all other experimental groups (Table 1; *p* = 0.001).

**Table 1:** Means (± standard deviations) of the hydrogen peroxide concentration (µg/mL) detected into the pulp chamber and initial concentration of hydrogen peroxide (%) in different experimental groups.

Experimental groups*	Initial concentration (%)	pH	HP concentration (μg/mL)
PolaDay (PD)	5.7 ± 0.1 b	5.9 ± 0.02 ^b^	0.027 ± 0.008 A,B
Pola Luminate (PL)	6.2 ± 0.1 a	5.7 ± 0.01 ^b^	0.016 ± 0.006 A
Viva Style (VS)	5.2 ± 0.2 b	4.9 ± 0.02 ^c^	0.035 ± 0.010 B
White Class (WC)	7.0 ± 0.1 a	5.5 ± 0.01 ^b^	0.017 ± 0.005 A
Whiteness Automixx (AM)	5.2 ± 0.2 b	7.9 ± 0.04 ^a^	0.029 ± 0.008 A,B
Control Group (CG)	-	-	0.000 ± 0.001**

(*) Same lower case, superscript or capital letters in each column indicate statistically similar means among groups (Tukey’s post-hoc test, p < 0.05).

(**) All experimental groups were statistically different when compared with the control group (Dunnett’s post-hoc test, p < 0.05).

### HP quantification inside the pulp cavity

The specimens showed standardized thicknesses with values varying between 3.1 and 3.5 mm (*p* = 0.52). Table 1 shows the amount of HP inside the pulp cavity. A lower amount of HP was found in the pulp cavity in the CG when compared to all experimental groups (Table 1; *p* < 0.000001). Regarding the different bleaching techniques evaluated, a significant difference was observed between groups (Table 1; *p* = 0.001). PL and WC showed a significantly lower amount of HP inside the pulp cavity when compared to VS. On the other side, PD and AM showed intermediary results when compared to all groups (Table 1; *p* > 0.05).

### Bleaching efficacy evaluation

The intra-examiner agreement level was 92% (Kappa statistics) when assessed color outcomes. Table 2 shows the bleaching efficacy measurements. A significant difference was found when comparing the CG with all experimental groups (*p* = 0.0001 for ΔE^*^_ab_, and *p* = 0.00001 for ΔE_00_; Table 2). Regarding the different bleaching techniques evaluated, no significant difference in terms of bleaching efficacy were observed when different groups were compared for ΔE^*^_ab_, (*p* = 0.38) and ΔE_00_ (*p* = 0.42). An example of bleaching specimens before and after bleaching can be seen in Figure 1.

**Table 2:** Means (± standard deviations) of the color change in different objective assessments (ΔE_ab*_ and ΔE_00_) in different experimental groups (*).

Experimental groups*	ΔE_ab*_	ΔE_00_
Pola Luminate (PL)	9.3 ± 2.7 A	5.6 ± 1.7 a
PolaDay (PD)	10.8 ± 2.9 A	6.8 ± 1.9 a
Viva Style (VS)	10.0 ± 3.6 A	5.7 ± 1.5 a
White Class (WC)	9.6 ± 3.4 A	5.3 ± 2.5 a
Whiteness Automixx (AM)	10.5 ± 3.8 A	5.9 ± 2.0 a
Control Group (CG)	2.2 ± 0.9 *	1.2 ± 0.5 *

(*) All experimental groups were statistically different when compared with the control group (Dunnett’s post-hoc test, p < 0.05).

(**) Same capital or lowercase letters in each column indicate statistically similar means among groups (Tukey’s post-hoc test, p < 0.05).

### Toxicity in Tenebrio molitor model

**Figure 2:**
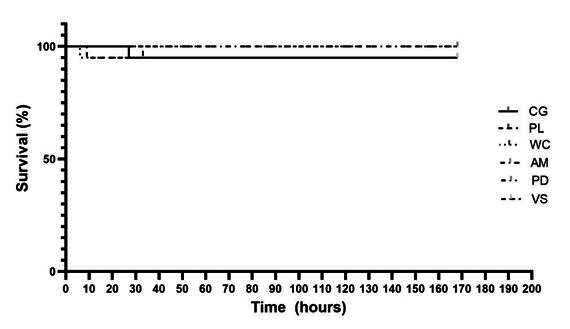
Kaplan-Meier survival rate of *T. molitor* larvae. CG = control group; PL = Pola Luminate; WC = White Class; AM = Whiteness HP Automixx; PD = Pola Day; VS = VivaStyle Paint On Plus.

The application of HP under the minimal initial concentration and 10 times of minimal initial did not show any toxicity to *T. molitor* larvae, resulting in a survival curve similar to that of *T. molitor* larvae in the CG for all experimental groups (Figure 2).

## Discussion

The present study evaluated the bleaching gels’ initial concentrations compared with the concentration the manufacturer indicated. Generally, the concentration observed in all products were slightly different when compared to those the manufacturer provided. This change in the products’ concentration seems to be common because previous studies have already reported an increase or decrease in the HP concentration of several bleaching products ^10^ ^,^ ^18^. However, in the present study, two bleaching products (PL and WC) showed a significantly higher HP available than other products did.

It was expected that the higher the amount of HP in the products, the higher the amount of HP inside the pulp chamber (8, 11). Interestingly, the present study did not observe these results. Actually, the bleaching gels with an increased amount of HP available (PL and WC) showed the lower amount of HP inside the pulp chamber, mainly when compared to VS. Other characteristics of bleaching gels could help to explain the present results. Observe that the most acidic bleaching gel-VS-showed the highest amount of HP inside the pulp chamber, despite the lower amount of HP available and the lower application time the manufacturer recommended (10min). This may be explained by the ability of the products’ pH to modulate the penetration of HP.

Most manufacturers of at-home bleaching agents present their products with an acidic pH to keep the products’ shelf life high ^19^. Despite that this seems beneficial for this purpose, unfortunately, the lower bleaching agent’s pH is associated with a higher amount of HP inside the pulp chamber when compared to less acidic or neutral pH bleaching gels ^20^ ^,^ ^21^. When more acidic pH bleaching gels are applied in the tooth surface, morphological alterations occur with the decreasing of the enamel hardness due to the superficial demineralization. These factors increase the tooth’s permeability, and consequently increase the amount of HP penetration inside the pulp chamber, as previously observed ^22^ ^,^ ^23^. In addition, bleaching gels with more acidic pH takes a longer time to complete the decomposition of HP when compared to less acidic bleaching gels ^24^. This means that the bleaching gels become active for more time, which increases the chance of HP to penetrate the pulp chamber.

It is worth mentioning that the sample size for this study was designed to detect a substantial difference among groups, specifically a 65% reduction in the amount of hydrogen peroxide inside the pulp chamber. Additionally, we observed considerable variability within each group, averaging 29%. This level of variability and sample size are consistent with those reported in previous studies ^9^ ^,^ ^11^ ^,^ ^12^ ^,^ ^13^. Future research with larger sample sizes is recommended to address these issues and provide more precise estimates.

However, despite these differences in the amount of HP penetration inside the pulp cavity among different products, the results of cytotoxicity in *T. molitor* larvae showed no statistical difference between them, even when 10 times of the minimal initial concentration was applied. This means that, despite significant differences, this may not be enough to cause toxicity in this in vitro model. T. molitor larvae provide valuable preliminary toxicity insights, aiding in drug development by excluding toxic compounds early in the process compared to more complex models ^17^.

To the extent of the authors' knowledge, this is the first study that uses this model of toxicity in larvae evaluating bleaching products. This model is simple, low-cost, fast, reproducible and does not require ethics committee approval when compared to other animal models ^17^ when compared to ISO 10993-12:2012. Unfortunately, considering this is the first study to use this methodology to evaluate the cytotoxicity of HP, it is impossible to evaluate if the survival rate in the larvae model can or cannot be correlated with tooth sensitivity the bleaching agents caused in a clinical study. Therefore, future studies need to be performed to evaluate this hypothesis, especially in at-home and in-office bleaching materials.

It is worth mentioning that only one in-office bleaching product was evaluated in the present study, mainly because no other low-concentrated in-office bleaching product was found in the market. AM is one in-office bleaching product presented in self-mixing, that is, the HP and the thickener are presented in separate tubes ^11^. This can benefit the bleaching agent’s pH because as a mixture is performed immediately before it is used, a more alkaline product is available for bleaching, which the present study’s results observed. However, the high pH of AM did not decrease the amount of HP inside the pulp chamber as expected. Actually, the longer application time indicated for this material can explain this. While the at-home bleaching gels were indicated for 10 to 30 min, the in-office bleaching gel was applied for 50 min. It has already been shown that the longer the application time, the greater the amount of HP inside the pulp ^4^ ^,^ ^9^.

Regarding color change, a significant bleaching efficacy was observed for all groups when compared to the CG, indicating that both parameters (ΔE^*^_ab_ or ΔE_00_) are sensible to evaluate the bleaching efficacy when compared to the CG. The ΔE^*^_ab_ is the most widely parameter used in bleaching studies ^24^, while the ΔE_00_, with a formula improvement ΔE^*^_ab_, is more faithful of color differences as the human eye can perceive them ^24^. For a procedure to be considered efficacy regarding color change, the changes must reach perceptible limits of ΔE^*^_ab_ > 2.7 and ΔE_00_ > 1.2 ^24^. When the different experimental groups were compared, no significance was observed among them when ΔE^*^_ab_ or ΔE_00_ were applied.

The variation between 9.3 to 10.8 for ΔE^*^_ab_ and 5.3 for 6.8 for ΔE_00_ in the bleaching efficacy observed in the present study was similar to those observed in the previous studies in which the same HP concentration was evaluated ^13^. It is worth mentioning that although a significant difference was observed in the initial concentration for the different bleaching gels evaluated, the difference in the amount of HP available for the different bleaching gels was not enough to show any significant difference regarding bleaching efficacy ^25^.

One of the current study's limitations was that all products were used according to the manufacturer’s recommendations. On one hand, this is crucial for assessing their behavior under real-world conditions. On the other hand, this means there were differences in the application mode and time because some bleaching gels were available in syringes and others in brushes. Furthermore, the application durations ranged from 10 to 50 minutes. However, it is possible to affirm that despite these differences, the bleaching gels with 6% HP evaluated in the present study demonstrated bleaching efficacy and minimal penetration into the pulp chamber. To validate the findings observed in this in vitro study, thoroughly delineated future randomized clinical trials are necessary.

## Conclusion

 Despite some punctual differences among bleaching gels based on 6% HP, all materials had bleaching efficacy, low penetration into the pulp chamber and were not toxic to T. *molitor* larvae.
